# Measures and models of visual acuity in epipelagic and mesopelagic teleosts and elasmobranchs

**DOI:** 10.1007/s00359-023-01661-7

**Published:** 2023-08-12

**Authors:** Eleanor M. Caves, Tracey T. Sutton, Eric J. Warrant, Sönke Johnsen

**Affiliations:** 1 Department of Ecology, Evolution, and Marine Biology, University of California Santa Barbara, Santa Barbara, CA 93106 USA; 2 Department of Marine and Environmental Sciences, Halmos College of Arts and Sciences, Nova Southeastern University, Dania Beach, FL 33004 USA; 3 Department of Biology, Lund University, Biology Building, Sölvegatan 35, Lund, Sweden; 4 Department of Biology, Duke University, Durham, NC 27708 USA

**Keywords:** Spatial resolution, Fish vision, Visual ecology, Light level, Deep sea

## Abstract

Eyes in low-light environments typically must balance sensitivity and spatial resolution. Vertebrate eyes with large "pixels" (e.g., retinal ganglion cells with inputs from many photoreceptors) will be sensitive but provide coarse vision. Small pixels can render finer detail, but each pixel will gather less light, and thus have poor signal relative-to-noise, leading to lower contrast sensitivity. This balance is particularly critical in oceanic species at mesopelagic depths (200–1000 m) because they experience low light and live in a medium that significantly attenuates contrast. Depending on the spatial frequency and inherent contrast of a pattern being viewed, the viewer’s pupil size and temporal resolution, and the ambient light level and water clarity, a visual acuity exists that maximizes the distance at which the pattern can be discerned. We develop a model that predicts this acuity for common conditions in the open ocean, and compare it to visual acuity in marine teleost fishes and elasmobranchs found at various depths in productive and oligotrophic waters. Visual acuity in epipelagic and upper mesopelagic species aligned well with model predictions, but species at lower mesopelagic depths (> 600 m) had far higher measured acuities than predicted. This is consistent with the prediction that animals found at lower mesopelagic depths operate in a visual world consisting primarily of bioluminescent point sources, where high visual acuity helps localize targets of this kind. Overall, the results suggest that visual acuity in oceanic fish and elasmobranchs is under depth-dependent selection for detecting either extended patterns or point sources.

## Introduction

**Fig. 1:**
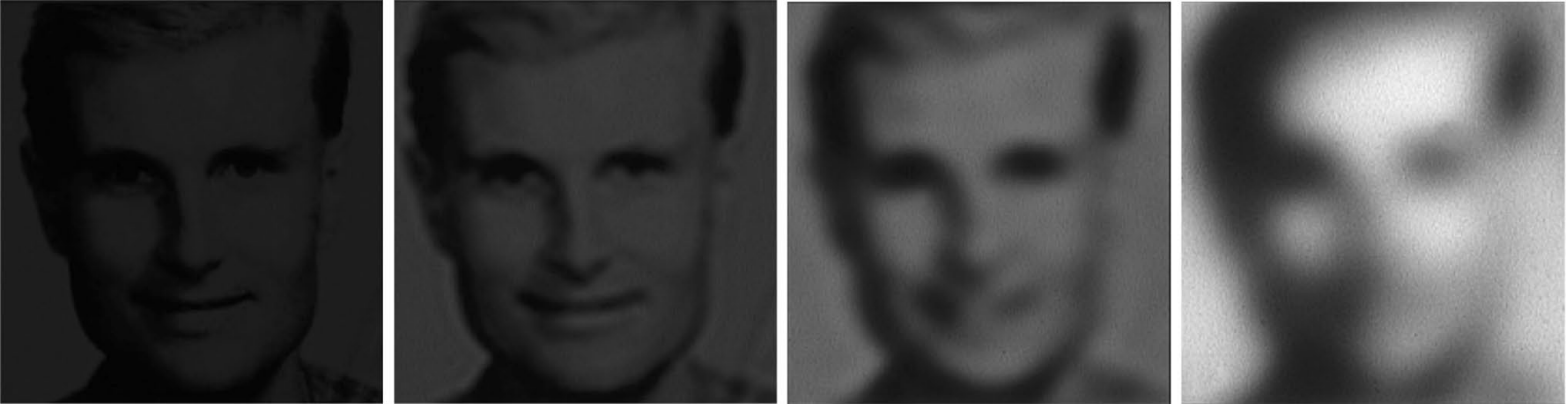
For a given amount of information, a system can either have few channels with high precision or many channels with low precision. For eyes, this trade-off manifests as a trade-off between resolution and sensitivity. Starting on the left, an eye can have high resolution but low sensitivity; moving to the right, sensitivity increases but at the expense of resolution

According to information theory (Shannon and Weaver 1949), the amount of information available to a receiving system depends on both the resolution of the system (i.e., number of channels that collect the information), and the reliability of the information in each channel (i.e., signal-to-noise ratio). Specifically, for a given amount of information, a system of a given size can possess either low resolution with high precision, or high resolution but low precision depending on how many channels it is divided into. Eyes, in which photons (information) must be collected by photoreceptive channels, are also subject to this trade-off. Thus, a retina with fewer channels that each collect information over a larger visual field has lower spatial resolution but higher signal-to-noise (and thus greater contrast sensitivity) in each channel (Fig. 1). The same retina, but with many channels, would potentially have higher resolution but at the cost of lower signal-to-noise in each channel because each channel collects fewer photons over a given integration time (Cronin et al. 2014). Because a system with zero or infinite channels collects zero information, and because information is non-negative, for any set of conditions there must exist an optimal density of channels for information transfer. In the case of vision, depending on the morphology and physiology of the eye, the optical environment, and the characteristics of the target, there exists a visual acuity of the viewer that allows the viewer to discern the critical features of the target at the greatest distance.

In vertebrate eyes, the light information gathered by each photoreceptor is sent via intermediate steps to a retinal ganglion cell (RGC), which in turn sends the combined inputs from some number of photoreceptors to the brain (Pettigrew et al. 1988; Lee and Stevens 2007). The number of photoreceptors that synapse with a given RGC thus determines the maximum resolution of the image in the brain, and each RGC can be considered a “pixel” of the perceived visual image. Although, in theory, one could decrease the diameter of the photoreceptors or pack them more tightly within the retina to affect resolution, in reality, vertebrate photoreceptors are typically already close-packed and their diameters are fairly conserved due to restrictions resulting from the wave nature of light (Cronin et al. 2014). Thus, it is the angular density of RGCs that typically determines visual acuity in vertebrate eyes.

In the daytime pelagic ocean, light intensity and the nature of visual scenes themselves vary predictably and dramatically with increasing depth. This has had a significant influence on visual system evolution in the organisms inhabiting these environments. In shallow water, light from the sun (and to a lesser extent the moon and stars) dominates, and the visual environment is one of extended scenes (Warrant 2000; Warrant and Locket 2004). Animals in this realm use their eyes for a variety of tasks, including orientation and locating and recognizing mates, predators, and prey. Thus, the visual acuity of eyes at epipelagic depths (0–200 m) and upper mesopelagic depths (200–600 m) may be specialized for discerning patterns on relevant targets (e.g., stripes on fishes). As depth increases, light dims exponentially, but scenes can still be considered extended or semi-extended. However, in dim enough environments, any eye with high resolution would likely have very low sensitivity and precision, not collecting enough photons for image creation under ambient light (Land 1990; Warrant and Locket 2004). Once greater mesopelagic depths (> 600 m) are reached, the majority of visual scenes become point-like, dominated by bioluminescent sources (Warrant 2000; Warrant and Locket 2004).

Given these depth-dependent differences in the fundamental nature of visual scenes and the associated differences in visual needs, whether and how visual acuity may be adapted for detecting details may vary with depth. Specifically, at shallow depths where the visual field comprises bright extended scenes, vision in diurnal fishes should be adapted for detecting detail; however, with increases in depth, eyes should begin to favor sensitivity, and resolution should drop. At depths and/or times of day where few to no scenes are extended, however, eyes should no longer be adapted for detecting patterns, and rather may exhibit increased acuity for more accurate localization of point sources (Wagner et al. 1998; Warrant 2000). Depth is not the only factor that can shift the nature of a visual scene, however, and in epipelagic and benthopelagic environments, light attenuation due to phytoplankton, dissolved organic matter, and other substances can impact a visual scene. Specifically, in high attenuation environments, the increasing absorption and scattering of light result in both attenuation of contrast at all spatial frequencies due to absorption and single scattering and additional attenuation of contrast at high spatial frequencies due to multiple scattering (Wells 1969; Gazey 1970); thus, in high attenuation environments, eyes are not predicted to be adapted for resolution, because the properties of the environment preclude those details being visible at distance regardless of the visual system employed.

In sum, for a given set of conditions comprising depth, turbidity, time of day, season, sea/sky state, etc., there exists a visual acuity that maximizes the viewing distance at which a given pattern can be detected. Here, we build an optical/visual model to examine the relationship between visual acuity and sighting distance in the open ocean and explore how this relationship depends on the optical properties of the object being sighted, the inherent optical properties of the water, and the amount of light available to the viewer’s retina—which in turn is a function of ambient light level, and the pupil area and temporal resolution of the viewer’s eye. We then use this model to calculate the predicted optimal visual acuity for a given animal at a given depth assuming an average daytime solar elevation, average oceanic cloud cover, and common values for turbidity and sea state (note that, although there are many ways that vision can be “optimal,” here and throughout we use the term to refer to the acuity value predicted by our optical model to maximize viewing distance). We then explore how these predicted acuities relate to a newly collated database of visual acuity in epipelagic and mesopelagic species of teleost fish and elasmobranchs.

## Methods

### Comparative database of acuity, lens diameter, body length, and habitat

We assembled a database of visual acuity in ray-finned fishes and elasmobranchs using published data. The true functional acuity of an animal is best measured using behavioral methods. However, in cases where animal behavior cannot be assessed (as in deep-sea fishes, which do not often survive long after capture), acuity can be estimated using a variety of methods, including by quantifying the peak (e.g., foveal) density of either photoreceptors or retinal ganglion cells (RGCs) (which often have a 1:1 relationship in areas of high density such as foveae, as in many teleost fishes, e.g., Fritsch et al. (2017)), or by measuring the optical quality of the lens. The photoreceptors, which in deep-sea fishes are typically only rods (Warrant and Lockett 2004), represent the sampling array of an eye, and thus may place an upper limit on acuity, although since numerous photoreceptors may connect to a single RGC, it has been suggested that the peak density of RGCs provides a more accurate estimate of acuity (Pettigrew et al. 1988). Lens optical quality sometimes yields higher estimates of acuity than other methods (e.g., Tamura 1957; Charman and Tucker 1973), though at least one study in pelagic fishes found that lens resolving power was a relatively good match for the resolution predicted by peak RGC density (Gagnon et al. 2013). Thus, given arguments in favor of each method, and the relative scarcity of literature on the visual acuities of deep-sea species, we included acuity estimates based on all three methods in our database to maximize sample size. In all cases, acuity was reported in units of either minutes of arc, minimum resolvable angle (MRA) in degrees, or in cycles per degree (cpd), which is the number of black and white stripe pairs an animal can resolve within a single degree of visual angle, and which is in the inverse of MRA in degrees. For this study, we converted all published values to cycles per degree. In two species, we found published acuity values from two different studies (Table 1); in these cases, the average of the two acuity values was used for analysis.

**Table 1:** Comparative database of acuity, lens diameter, habitat, and depth ranges in elasmobranchs and teleosts

	Binomial	Acuity measurement method	Acuity source	Acuity (°)	Lens diameter (mm)	Habitat	In/under productive water?	Min –Max depth (m)	Depth source
*Elasmobranchs*	*Alopias superciliosus*	RGC density	(Lisney and Collin 2008)	0.10	30.3	Epipelagic	No	0–100	(Froese and Pauly 2000)
	*Callorhinchus milii*	RGC density	(Garza-Gisholt et al. 2018)	0.08	9.86	Shallow demersal	Yes	0–227	(Froese and Pauly 2000)
	*Carcharhinus amblyrhynchos*	RGC density	(Lisney and Collin 2008)	0.39	12.7	Coastal pelagic	Yes	0–280	TS
	*Carcharhinus leucas*	RGC density	(Lisney and Collin 2008)	0.33	6	Coastal pelagic	Yes	0–50	(Froese and Pauly 2000)
	*Carcharhinus melanopterus*	RGC density	(Lisney and Collin 2008)	0.27	6.8	Benthopelagic	No	20–75	(Froese and Pauly 2000)
	*Carcharhinus plumbeus*	RGC density	(Litherland et al. 2009)	0.11	11.5	Benthopelagic	Yes	20–65	(Froese and Pauly 2000)
	*Centroscymnus coelolepis*	RGC density	(Bozzano 2004)	0.14	11.5	Bathydemersal	No	400–1750	(Froese and Pauly 2000)
	*Chimaera lignaria*	RGC density	(Garza-Gisholt et al. 2018)	0.34	21.7	Bathydemersal	No	800–1800	(Froese and Pauly 2000)
	*Etmopterus spinax*	RGC density	(Bozzano and Collin 2000)	0.36	8.4	Bathydemersal	No	200–500	(Froese and Pauly 2000)
	*Galeocerdo cuvieri*	RGC density	(Bozzano and Collin 2000)	0.03	13.7	Coastal and Epipelagic	Yes	0–50	TS
	*Galeus melastomus*^*a*^	RGC density	(Bozzano and Collin 2000)	0.24	7.3	Demersal	Yes	300–800	From *G. arae*; TS
	*Prionace glauca*	RGC density	(Lisney and Collin 2008)	0.44	15.2	Epipelagic	No	0–50	(Froese and Pauly 2000)
	*Rhinochimaera pacifica*	RGC density	(Garza-Gisholt et al. 2018)	0.38	14.0	Bathydemersal	No	330–1490	(Froese and Pauly 2000)
	*Scyliorhinus canicula*	RGC density	(Bozzano and Collin 2000)	0.13	6.8	Bathydemersal	Yes	80–100	(Froese and Pauly 2000)
	*Scyliorhinus torazame*	RGC density	(Muguruma et al. 2013)	0.33	5.3	Demersal	Yes	80–100	(Froese and Pauly 2000)
	*Sphyrna lewini*	RGC density	(Lisney and Collin 2008)	0.10	6.58	Coastal and bathydemersal	No	0–25	(Froese and Pauly 2000)
	*Sphyrna mokarran*	RGC density	(Lisney and Collin 2008)	0.42	14.5	Coastal and epipelagic	Yes	1–100	(Froese and Pauly 2000)
	*Sphyrna zygaena*	RGC density	(Lisney and Collin 2008)	0.18	11.8	Coastal and epipelagic	yes	0–20	(Froese and Pauly 2000)
	*Squalus mitsurkurii*	RGC density	(Litherland et al. 2009)	0.14	12.5	Benthopelagic	Yes	48–533	(Froese and Pauly 2000)
*Teleosts*	*Acanthocybium solandri*	Cone density	(Tamura and Wisby 1963)	0.04	18.5	Pelagic-oceanic	No	0–12	(Froese and Pauly 2000)
	*Acanthopagrus berda*	Cone density	(Tamura 1957)	0.10	6.0	Demersal	Yes	0–20	(Froese and Pauly 2000)
	*Alepocephalus bairdii*	RGC density	(Wagner et al. 1998)	0.04	11.7	Bathydemersal	No	1000–1700	(Froese and Pauly 2000)
	*Alepocephalus rostratus*	RGC density	(Collin and Partridge 1996; Wagner et al. 1998)	0.08	3.4	Bathydemersal	No	1000–2250	(Froese and Pauly 2000)
	*Anoplogaster cornuta*	Lens optics	(Gagnon et al. 2013)	0.04	2.6	Meso-to-Bathypelagic	No	500–2000	(Froese and Pauly 2000)
	*Argyropelecus aculeatus*	Lens optics	(Gagnon et al. 2013)	0.08	3.4	Mesopelagic	No	300–600	(Baird 1971; Badcock 1984)
	*Argyropelecus affinis*	RGC density	(Wagner et al. 1998)	0.10	3.8	Mesopelagic	No	300–600	(Baird 1971; Badcock 1984)
	*Argyropelecus gigas*	RGC density	(Wagner et al. 1998)	0.12	4.4	Mesopelagic	No	300–650	(Baird 1971; Badcock 1984)
	*Argyropelecus hemigymnus*	RGC density	(Wagner et al. 1998)	0.13	2.4	Mesopelagic	No	300–600	(Baird 1971; Howell and Krueger 1987)
	*Argyropelecus sladeni*	RGC density	(Collin and Partridge 1996; Wagner et al. 1998)	0.13	3.05	Mesopelagic	No	350–650	(Schultz 1964; Baird 1971)
	*Astronesthes lucifer*	Lens optics	(Gagnon et al. 2013)	0.12	2.8	Mesopelagic	No	185–560	(Gagnon et al. 2013)
	*Avocettina infans*	Lens optics	(Gagnon et al. 2013)	0.20	1.84	Meso-to-Bathypelagic	No	1200–2000	(Gagnon et al. 2013)
	*Bathytroctes microlepis*	RGC density	(Collin and Partridge 1996; Wagner et al. 1998)	0.17	3.48	Bathydemersal	No	1900–4900	(Markle and Quéro 1984; Anderson et al. 1985)
	*Benthosema suborbitale*	Lens optics	(Gagnon et al. 2013)	0.12	3	Mesopelagic	No	375–750	(Gartner et al. 1987)
	*Bolinichthys longipes*	RGC density	(de Busserolles et al. 2014)	0.12	1.8	Mesopelagic	No	425–750	(Nafpaktitis et al. 1977)
	*Bolinichthys nikolayi*^*a*^	RGC density	(de Busserolles et al. 2014)	0.07	1.8	Mesopelagic	No	425–750	from B*. longipes*
	*Ceratoscopelus warmingii*	RGC density	(de Busserolles et al. 2014)	0.40	1.5	Mesopelagic	No	425–1100	(Hulley 1984; Gartner et al. 1987)
	*Chauliodussloani*	Lens optics	(Gagnon et al. 2013)	0.10	2.8	Meso-to-bathypelagic	No	494–1000	(Gage and Tyler 1991; Shinohara et al. 1994; Mundy 2005)
	*Clupea harengus*	Cone density	(Blaxter and Jones 1967)	0.24	7	Coastal and epipelagic	Yes	0–20	TS
	*Coccorella atlantica*	Lens optics	(Gagnon et al. 2013)	0.27	2.8	Mesopelagic	No	500–1000	(Gagnon et al. 2013)
	*Cololabis saira*	Cone density	(Hajar et al. 2008)	0.11	4.5	Coastal and epipelagic	Yes	0–230	(Froese and Pauly 2000)
	*Conocara macroptera*	RGC density	(Wagner et al. 1998)	0.14	8.5	Bathydemersal	No	1200–1800	(Wagner et al. 1998; Froese and Pauly 2000)
	*Conocara salmoneum*	RGC density	(Wagner et al. 1998)	0.50	13.5	Bathydemersal	No	3000–4500	(Wagner et al. 1998; Froese and Pauly 2000)
	*Coryphaena hippurus*	Cone density	(Tamura and Wisby 1963)	0.23	5	Epipelagic	No	0–20	TS
	*Dasyscopelus asper*^*b*^	RGC density	(de Busserolles et al. 2014)	0.11	3.7	Mesopelagic	No	425–750	(Nafpaktitis 1977)
	*Dasyscopelus obtusirostris*^*b*^	RGC density	(de Busserolles et al. 2014)	0.34	4.6	Mesopelagic	No	325–750	(Nafpaktitis et al. 1977)
	*Dasyscopelus spinosus*^*b*^	RGC density	(de Busserolles et al. 2014)	0.26	2.5	Mesopelagic	No	0–700	(Froese and Pauly 2000)
	*Dentex tumifrons*	Cone density	(Tamura 1957)	0.11	4.0	Demersal	Yes	30–346	(Froese and Pauly 2000)
	*Diaphus mollis*	RGC density	(de Busserolles et al. 2014)	0.12	3.2	Mesopelagic	No	300–800	(Nafpaktitis 1978; Gartner et al. 1987)
	*Diaphus parri*^*a*^	RGC density	(de Busserolles et al. 2014)	0.34	1.8	Mesopelagic	No	300–800	From *D. mollis*
	*Diogenichthys laternatus*^*a*^	RGC density	(de Busserolles et al. 2014)	0.12	0.7	Mesopelagic	No	450–120	from *D. atlanticus*; (Hulley 1984)
	*Diplospinus multistriatus*	Optics	(Gagnon et al. 2013)	0.21	2.2	Benthopelagic	No	500–1000	(Gagnon et al. 2013)
	*Dolichopteryx sp*	RGC density	(Wagner et al. 1998)	0.10	1.9	Mesopelagic	No	0–1000	(Cohen 1964)
	*Electrona risso*	RGC density	(de Busserolles et al. 2014)	0.07	3.9	Mesopelagic	No	225–750	(Nafpaktitis et al. 1977)
	*Glossanodon semifasciatus*^*a*^	Cone density	(Tamura 1957)	0.15	5.2	Benthopelagic	Yes	180–400	From *G. pygmaeus;* (McEachran and Fechhelm 1998)
	*Idiacanthus antrostomus*	Optics	(Gagnon et al. 2013)	0.33	1.94	Meso-to-bathypelagic	No	500–2000	(Gagnon et al. 2013)
	*Istiophorus albicans*	Cone density	(Tamura and Wisby 1963)	0.21	11.4	Epipelagic	No	0–50	(Froese and Pauly 2000)
	*Jaydia lineata*^*b*^	Cone density	(Tamura 1957)	0.24	4.3	Demersal	Yes	1–100	(Froese and Pauly 2000)
	*Kajikia albida*	Cone density	(Tamura and Wisby 1963)	0.37	14.5	Epipelagic	No	0–100	(Froese and Pauly 2000)
	*Katsuwonus pelamis*	Cone density	(Tamura and Wisby 1963)	0.11	17	Epipelagic	No	0–50	(Froese and Pauly 2000)
	*Lampadena luminosa*	RGC density	(de Busserolles et al. 2014)	0.32	3.7	Mesopelagic	No	425–850	(Hulley 1986; Gartner et al. 1987)
	*Lampanyctus alatus*	RGC density	(de Busserolles et al. 2014)	0.36	1.3	Mesopelagic	No	275–1000	(Hulley 1984; Gartner et al. 1987)
	*Lampanyctus ater*^*b*^	RGC density	(Wagner et al. 1998)	0.63	1.15	Mesopelagic	No	550–1850	(Nafpaktitis et al. 1977)
	*Lampanyctus festivus*	RGC density	(Wagner et al. 1998)	0.21	2.3	Mesopelagic	No	750–950	(Badcock 1970)
	*Lampanyctus parvicauda*	RGC density	(de Busserolles et al. 2014)	0.21	1.4	Mesopelagic	No	100–500	(Froese and Pauly 2000)
	*Leiognathus equulus*	Cone density	(Tamura 1957)	0.19	4.5	Demersal	No	10–110	(Froese and Pauly 2000)
	*Lepidophanes guentheri*	Optics	(Gagnon et al. 2013)	0.28	3	Mesopelagic	No	400–900	(Gagnon et al. 2013)
	*Makaira nigricans*	RGC density	(Fritsches et al. 2003)	0.36	19	Epipelagic	No	0–50	TS
	*Malacosteus niger*	Optics	(Gagnon et al. 2013)	0.16	3.2	Bathypelagic	No	500–900	(Gagnon et al. 2013)
	*Malakichthys wakiyae*	Cone density	(Tamura 1957)	0.24	4	Coastal and Epipelagic	Yes	100–200	(Froese and Pauly 2000)
	*Melanolagus bericoides*	Optics	(Gagnon et al. 2013)	0.15	5.2	Meso-to-Bathypelagic	No	750–1700	(Gagnon et al. 2013)
	*Myctophum brachygnathum*	RGC density	(de Busserolles et al. 2014)	0.18	3.3	Mesopelagic	No	280–340	(de Busserolles et al. 2014)
	*Myctophum lychnobium*	RGC density	(de Busserolles et al. 2014)	0.32	2.3	Mesopelagic	No	0–1000	(Froese and Pauly 2000)
	*Myctophum nitidulum*	RGC density	(de Busserolles et al. 2014)	0.16	3.1	Mesopelagic	No	475–850	(Nafpaktitis et al. 1977)
	*Narcetes stomias*	RGC density	(Wagner et al. 1998)	0.03	6.3	Bathydemersal	No	1800–2100	(Wagner et al. 1998; Froese and Pauly 2000)
	*Notoscopelus kroeyerii*	RGC density	(de Busserolles et al. 2014)	0.24	2.7	Mesopelagic	Yes	800–900	(Froese and Pauly 2000)
	*Opisthoproctus grimaldii*	RGC density	(Wagner et al. 1998)	0.03	2.2	Mesopelagic	No	200–600	(Cohen 1964)
	*Opisthoproctus soleatus*^*c*^	Lens optics/RGC density	(Gagnon et al. 2013; Wagner et al. 1998)	0.04/0.20	5.4	Mesopelagic	No	200–600	(Cohen 1964)
	*Platytroctes apus*	RGC density	(Collin and Partridge 1996; Wagner et al. 1998)	0.36	5.1	Bathypelagic	No	1000–5393	(Quéro 1984)
	*Regalecus glesne*	Lens Optics	(Gagnon et al. 2013)	0.77	1.28	Pelagic-oceanic	No	20–200	(Froese and Pauly 2000; Gagnon et al. 2013)
	*Rouleina attrita*	RGC density	(Collin and Partridge 1996; Wagner et al. 1998)	0.50	3.43	Meso-to-bathypelagic	No	1400–2100	(Markle and Quéro 1984)
	*Scomber australasicus*	Cone density	(Kawamura 1979)	0.39	8.84	Costal pelagic	Yes	0–200	(Froese and Pauly 2000)
	*Scomber japonicus*	Cone density	(Tamura 1957)	0.19	4.6	Coastal pelagic	Yes	50–200	(Froese and Pauly 2000)
	*Scopelarchus michaelsarsi*	RGC density	(Collin and Partridge 1996)	0.31	2.25	Mesopelagic	No	250–500	(Johnson 1974, 1986)
	*Scopeloberyx robustus*	Lens optics	(Gagnon et al. 2013)	0.12	2.6	Meso-to-bathypelagic	No	750–2300	(Gagnon et al. 2013)
	*Scopelosaurus hoedti*	Lens optics	(Gagnon et al. 2013)	0.18	3.2	Mesopelagic	No	300–600	(Gagnon et al. 2013)
	*Searsia koefoedi*	RGC density	(Collin and Partridge 1996; Wagner et al. 1998)	0.20	4.25	Bathypelagic	No	450–1500	(Froese and Pauly 2000)
	*Selar crumenophthalmus*	Lens optics	(Gagnon et al. 2013)	0.26	3.8	Coastal pelagic	yes	0–50	(Gagnon et al. 2013)
	*Seriola lalandi*	RGC density	(Nagloo et al. 2017)	0.14	7.0	Benthopelagic	Yes	0–100	(Froese and Pauly 2000)
	*Seriola quinqueradiata*^*c*^	Cone density/cone density	(Tamura 1957; Miyagi et al. 2001)	0.42/0.23	3.2	Coastal pelagic	Yes	0–100	(Froese and Pauly 2000)
	*Sigmops elongatus*^*b*^	Lens optics	(Gagnon et al. 2013)	0.35	2	Mesopelagic	No	500–1200	(Badcock 1984)
	*Sternoptyx diaphana*	Lens optics	(Gagnon et al. 2013)	0.09	2.4	Meso-to-bathypelagic	No	700–1000	(Badcock and Baird 1980; Howell and Krueger 1987)
	*Stylephorus chordatus*	RGC density	(Wagner et al. 1998)	0.49	4.9	Meso-to-Bathypelagic	No	625–800	(Clarke 1976; Froese and Pauly 2000)
	*Symbolophorus rufinus*	RGC density	(de Busserolles et al. 2014)	0.26	2.8	Mesopelagic	No	425–875	(Nafpaktitis 2000)
	*Thunnus albacares*	Cone density	(Tamura and Wisby 1963)	0.11	11.2	Epipelagic	No	1–100	(Froese and Pauly 2000)
	*Trachurus japonicus*	Cone density	(Tamura 1957)	0.15	5.5	Coastal pelagic	Yes	0–50	(Froese and Pauly 2000)
	*Trichiurus lepturus*	Cone density	(Kawamura and Ohashi 1988)	0.08	5.7	Benthopelagic	Yes	100–350	(Froese and Pauly 2000)
	*Xenodermichthys copei*	RGC density	(Collin and Partridge 1996; Wagner et al. 1998)	0.17	1.84	Mesopelagic	No	100–1230	(Froese and Pauly 2000)

*TS* indicates depth source was supplied by co-author Tracey Sutton

^a^Depth data extrapolated from congener

^b^Indicates original citation listed different Latin name

^c^Indicates a species in which two acuity measures were found in the literature, and both are indicated in the table

To parameterize the model, we then also gathered information on lens diameter (as a proxy for pupil diameter), daytime depth range, and habitat type for each species in the acuity database. Daytime depth ranges were gathered from a combination of sources, including Fishbase.org (Froese and Pauly 2000) and primary literature (Table 1). Minimum and maximum daytime depths were recorded unless a “usual” depth range has been reported, and a species’ mid-depth was calculated as the midpoint between minimum and maximum depth, or the midpoint of the usual depth range, if known. In cases where no depth records could be found, depth ranges were informed by published depth ranges from a congener (*n* = 5).

Habitat type was assigned based on information from Fishbase.org and from an extensive vertical distribution database for a well-studied open-ocean system (Gulf of Mexico), which allowed us to classify species as bathydemersal, bathypelagic, benthopelagic, demersal, epipelagic, epi-to-mesopelagic, meso-to-bathypelagic, mesopelagic, coastal pelagic, or in some cases, a combination of designations for wide-ranging species. Benthic species (*n* = 6) were excluded from the analysis, given that water in these habitats can be significantly more turbid than that in the open-water column. Lastly, given that primary productivity in the epipelagic zone (0–200 m) can affect light levels within this zone and below it, and thus predicted optimal acuity, species were classified as living “in or under productive water” based on environmental classifications. For example, coastal and shelf/slope species were considered to be living in or under productive water, while offshore species typically were not. The final database contained data for 97 species (Table 1).

Where given, we also noted the total length of the individuals used in acuity analyses, as a measure of body length; where not given in the literature, length values were taken from FishBase.org. Total length is a straight-line measure of length from the tip of the snout to the tip of the longer lobe of the caudal fin; however, total length is only one potential measure of body length in a fish. In cases where only fork length or standard length measurements were available, we used species-specific conversions between length types given on FishBase.org to calculate total length for each species.

### Model of optimal visual acuity

**Fig. 2:**
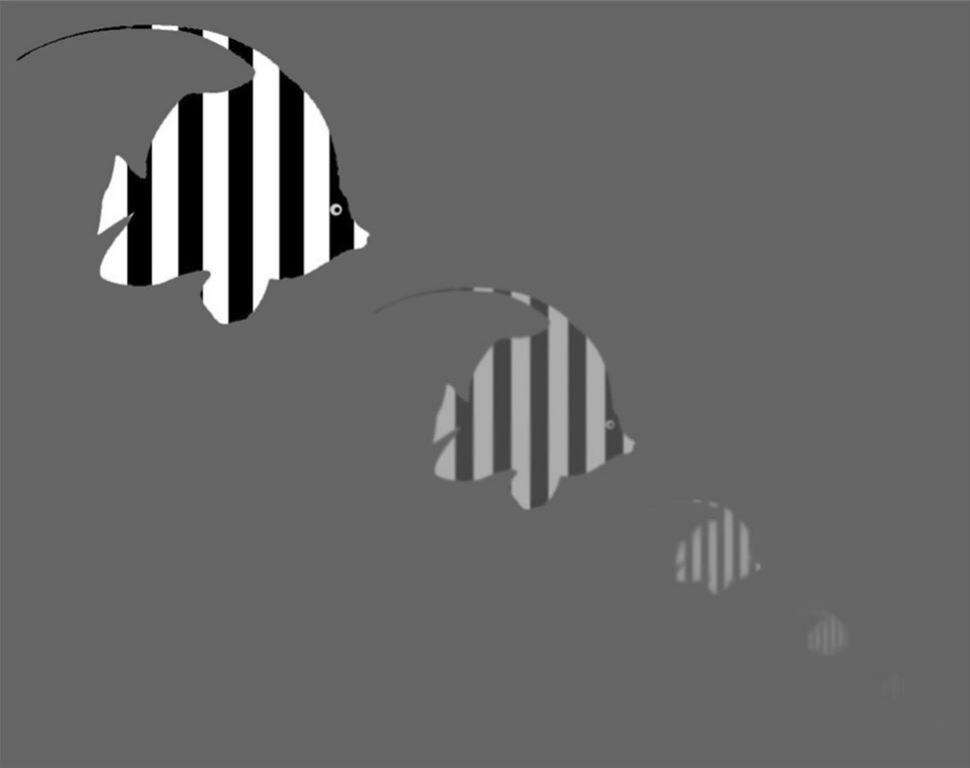
The effect of viewing distance on the size, contrast, and sharpness of a patterned target. As the distance between the viewer and the target increases, the target becomes smaller, reduces in contrast due to path light, and loses fine spatial detail due to multiple scattering. At a certain maximal distance, the stripes are just barely discernible

Although there are a number of ways to estimate the ability of a visual system to extract visual information from the environment, here we consider the maximum distance at which a pattern on a target viewed against a featureless water background can still be discerned, since this can be a relevant task for pelagic predators and prey. Consider a small, vertically oriented surface (e.g., lateral side of a fish) patterned with stripes that have a spatial period (width of two stripes) *s* that is viewed horizontally underwater at a distance *d*. Assume that *d* >  > *s*, so the angular period of the stripes (in radians) is well-approximated by *s*/*d*. For simplicity, we assume that the average reflectance of the light and dark stripes combined is always 50%. Assume for the moment that the water between the viewer and the target has no effect on the propagation of the image of the stripes. Then the Michelson contrast *C*(*d*) of the stripes when viewed by an animal with a minimum resolvable angle (inverse of spatial acuity) of $\Delta \rho$ is:

1 $$C\left( d \right) = C_{0} e^{{ - \gamma \left( {\tfrac{\Delta \rho d}{s}} \right)^{2} }} ,$$

where *C*_0_ is the inherent Michelson contrast of the stripes (contrast at zero distance) and $\gamma = 3.56$ (Snyder 1975). This drop in contrast is due to the fact that the stripes appear smaller with increasing distance, and the perceived contrast of smaller stripes is less than that of larger stripes (Fig. 2). At the maximum sighting distance of the stripes, by definition their perceived contrast equals the minimum contrast threshold of the viewer:

2 $$C_{\min } = C_{0} e^{{ - \gamma \left( {\tfrac{\Delta \rho d}{s}} \right)^{2} }} .$$

This minimum contrast threshold, however, depends on the visual acuity of the viewer. Suppose the visual system is viewing a striped target and an RGC collects $N_{0} - \tfrac{\varepsilon }{2}$ photons from the darker stripe. Then some neighboring RGC collects $N_{0} + \tfrac{\varepsilon }{2}$ photons from the lighter stripe, assuming the stripes are not too small to be resolved at the relevant distances (which is true in this study). *N*_0_ is the average number of photons the two RGCs see, and $\varepsilon$ is the difference in photons between the light and dark stripe*.* So, by definition, the Michelson contrast of the stripes is:

3 $$C = \frac{{\left( {N_{0} + \tfrac{\varepsilon }{2}} \right) - \left( {N_{0} - \tfrac{\varepsilon }{2}} \right)}}{{\left( {N_{0} + \tfrac{\varepsilon }{2}} \right) + \left( {N_{0} - \tfrac{\varepsilon }{2}} \right)}} = \frac{\varepsilon }{{2N_{0} }}.$$

The smallest contrast that can be reliably detected can be determined from the fact that the difference between the photon catches of the light and dark stripe is just detectable when it equals the square root of the sum of the catches multiplied by a reliability coefficient R (Nilsson et al. 2012), i.e., at the limit of detection:

4 $$\left( {N_{0} + \tfrac{\varepsilon }{2}} \right) - \left( {N_{0} - \tfrac{\varepsilon }{2}} \right) = \varepsilon = R\sqrt {\left( {N_{0} + \tfrac{\varepsilon }{2}} \right) + \left( {N_{0} - \tfrac{\varepsilon }{2}} \right)} = R\sqrt {2N_{0} } .$$

The reliability coefficient for 95% confidence is 1.96. Approximating this as 2, we get:

5 $$\varepsilon \cong 2\sqrt {2N_{0} } .$$

Substituting (5) into (3), we get the following for the minimum contrast threshold:

6 $$C_{\min } \cong \frac{1}{{\sqrt {2N_{0} } }},$$

where (Warrant 2006):

7 $$N_{{0}} = 1.13\frac{\pi }{4}\left( {\Delta \rho } \right)^{2} D^{2} \kappa \tau \Delta t\int {\left( {1 - e^{{ - kR_{i} \left( \lambda \right)l}} } \right)L_{{{\text{avg}}}} \left( \lambda \right)} d\lambda .$$

*N*_*0*_ is the number of photons that are absorbed by a single RGC looking at a stripe with 50% reflectance which has a radiance *L*_avg_(*λ*) over its integration time $\Delta t$. Other parameters include pupil diameter *D*, photoreceptor outer-segment length *l*, the minimum resolvable angle (MRA) $\Delta \rho$ (in radians), the quantum efficiency of transduction $\kappa$, the transmission of the lens/cornea/humors $\tau$, and the absorption coefficient of the photoreceptor *k*. The integral term describes the number of photons that will be absorbed in a photoreceptor of spectral sensitivity $R_{i} \left( \lambda \right)$. The terms before the integral determine the number of photons that the optics of the eye allows to reach the photoreceptors. $R_{i} \left( \lambda \right)$ in this case is calculated using the Stavenga–Smits–Hoenders rhodopsin template (Stavenga et al. 1993) with a given peak spectral sensitivity. Assuming that the target reflects light diffusely, the average radiance of the stripes is:

8 $$L_{{{\text{avg}}}} \left( \lambda \right) = \frac{{E_{h} \left( \lambda \right)}}{2\pi },$$

where $E_{h} \left( \lambda \right)$ is the side-welling irradiance (Johnsen 2002). $E_{h} \left( \lambda \right) = 10L_{h} \left( \lambda \right)$ at the dominant wavelength of 480 nm at depth, where $L_{h} \left( \lambda \right)$ is the horizontal background radiance (Johnsen 2002), so: $L_{{{\text{avg}}}} \left( \lambda \right) = \frac{{5L_{h} \left( \lambda \right)}}{\pi }$. Thus:

9 $$C_{\min } = \frac{1}{{\sqrt {2\left( {\Delta \rho } \right)^{2} \beta } }} = \frac{1}{{\Delta \rho \sqrt {2\beta } }},$$

where

10 $$\beta = \frac{{N_{0} }}{{\left( {\Delta \rho } \right)^{2} }} = 1.4D^{2} \Delta t\kappa \tau \int {\left( {1 - e^{{ - kR_{i} \left( \lambda \right)l}} } \right)L_{{\text{h}}} \left( \lambda \right)} d\lambda .$$

The term $\beta$, which pulls out the square of the visual acuity of the viewer, can be thought of as the photon catch per steradian of a single RGC. Substituting (9) into (2) gives:

11 $$\frac{1}{{\Delta \rho \sqrt {2\beta } }} = C_{0} e^{{ - \gamma \left( {\tfrac{\Delta \rho d}{s}} \right)^{2} }} .$$

However, the water itself does in fact reduce the contrast of the viewed image (Fig. 2). This effect can be represented as the product of two modulation transfer functions (MTFs). The first describes the equal reduction of contrast at all spatial frequencies due to the absorption and single scattering of light from the target by the medium and its replacement by light that is scattered into the path between the target and the viewer. The second function depends on spatial frequency (e.g., the perceived width of the stripes) and describes the effect of both multiple scattering and small spatial variations in the refractive index of the water on the contrast of finer detail. This second function is normalized to unity at zero spatial frequency. Thus, Eq. (2) must be rewritten as:

12 $$C_{\min } = C_{0} e^{{ - \gamma \left( {\tfrac{\Delta \rho d}{s}} \right)^{2} }} \cdot MTF_{w}^{1} \cdot MTF_{w}^{2} ,$$

where $MTF_{w}^{1}$ is the first, frequency-independent function and $MTF_{w}^{2}$ is the second frequency-dependent one (both depend on viewing distance and water clarity). This division is analogous to dividing the absorption of light in photoreceptors into a wavelength-independent absorption coefficient and a normalized wavelength-dependent absorbance function.

We first calculate $MTF_{w}^{1}$. Suppose the stripes have radiances of *L*_max_ and *L*_min_, and the water has a beam attenuation coefficient of the water of *c* and the same background horizontal radiance of *L*_h_. Then we get the following for Michelson contrast of the stripes *C* as a function of viewing distance:

13 $$C\left( d \right) = \frac{{\left( {L_{{\max }} e^{{ - cd}} + L_{{\text{h}}} \left( {1 - e^{{ - cd}} } \right)} \right) - \left( {L_{{\min }} e^{{ - cd}} + L_{{\text{h}}} \left( {1 - e^{{ - cd}} } \right)} \right)}}{{\left( {L_{{\max }} e^{{ - cd}} + L_{{\text{h}}} \left( {1 - e^{{ - cd}} } \right)} \right) + \left( {L_{{\min }} e^{{ - cd}} + L_{{\text{h}}} \left( {1 - e^{{ - cd}} } \right)} \right)}}.$$

Grouping terms and dividing the top and bottom by $\left( {L_{\max } + L_{\min } } \right)e^{ - cd}$ gives:

14 $$C\left( d \right) = C_{0} \left( {1 + \frac{{L_{{\text{h}}} }}{{L_{{{\text{avg}}}} }}\left( {e^{cd} - 1} \right)} \right)^{ - 1} = C_{0} \left( {1 + \frac{\pi }{5}\left( {e^{cd} - 1} \right)} \right)^{ - 1} ,$$

where *L*_avg_ is again the average radiance of the combined stripes (Cronin et al. 2014). Thus:

15 $$MTF_{w}^{1} = \left( {1 + \frac{\pi }{5}\left( {e^{cd} - 1} \right)} \right)^{ - 1} .$$

The normalized, frequency-dependent *MTF* of seawater has been determined by Ronald et al. (1969) to have the form:

16 $$MTF_{w}^{2} = e^{{ - \omega \left( {cd} \right)\tfrac{d}{s}}} ,$$

where $\omega$ depends on water type and on the product of viewing distance *d* and the beam attenuation coefficient *c*. For water types examined in this study, where scattering accounts for roughly half the total attenuation, $\omega$ is approximately:

17 $$\omega \left( {cd} \right) = \frac{cd}{{40}},$$

so

18 $$MTF_{w}^{2} = e^{{ - \tfrac{{cd^{2} }}{40s}}} .$$

Substituting (9), (15), and (18) into (12) gives:

19 $$\frac{1}{{\Delta \rho \sqrt {2\beta } }} = C_{0} e^{{ - \gamma \left( {\tfrac{\Delta \rho d}{s}} \right)^{2} }} \left( {1 + \frac{\pi }{5}\left( {e^{cd} - 1} \right)} \right)^{ - 1} e^{{ - \tfrac{{cd^{2} }}{40s}}} .$$

Inverting both sides and re-arranging gives:

20 $$C_{0} \sqrt {2\beta } \Delta \rho = e^{{\gamma \left( {\tfrac{\Delta \rho d}{s}} \right)^{2} }} \left( {1 + \frac{\pi }{5}\left( {e^{cd} - 1} \right)} \right)e^{{\tfrac{{cd^{2} }}{40s}}} ,$$

which must be solved numerically. The solution gives the relationship between the minimum resolvable angle of the viewer $\Delta \rho$ (which again is the inverse of visual acuity) and the sighting distance *d*, allowing one to find the visual acuity at which the stripes can be discerned at the greatest distance.

### Parameters for model

The background horizontal radiance spectra $L_{h} \left( \lambda \right)$ were modeled using commercial radiative transfer software (HydroLight 5.1, Sequoia Scientific). The ability of radiative transfer theory to accurately model oceanic radiance distributions has been validated by in situ measurements of selected radiances and irradiances in multiple studies (Mobley et al. 1993; Stramska et al. 2000). The agreement between modeled and measured spectral radiances is particularly good in oceanic waters, which are more easily characterized (reviewed by Mobley 1994).

Two water types were modeled. The first was “average ocean,” which was considered to be a modified “Case I” model (absorption and scattering dominated by chlorophyll and the water itself; Mobley 1994) with a Chl-a concentration of 0.2 mg/m^3^ at depths of 0–100 m and 0 mg/m^3^ at depths > 150 m, with a linear decrease in Chl-a concentration from 0.2 to 0 at depths of 100–150 m. The second water type was “productive ocean.” This was also a modified “Case I” model, but with a Chl-a concentration of 0.4 mg/m^3^ at depths of 0–100 m, 0 mg/m^3^ at depths > 150 m, with a linear decrease in Chl-a concentration from 0.4 to 0 at depths of 100–150 m. Average ocean is a proxy for offshore region that were not northern polar; “productive ocean” is a proxy for coastal/shelf/slope and northern polar regions. In both cases, the optical properties at depths > 150 m are considered to be dominated by absorption and scattering by the water itself.

Underwater radiance distributions were calculated from 400 to 700 nm at 10-nm intervals and from the surface to 1000 m depth at 50-m intervals. The sky was assumed to be 50% cloudy, the wind at 10 kts, and the sun at 30° elevation above the horizon—all chosen because they represent roughly average daytime values for the open ocean. The sky irradiance was calculated using the Radtran model (Gregg and Carder 1990), and the sky radiance angular distribution was calculated using the semi-empirical model given in Harrison and Coombes (1988). Both models account for atmospheric effects, such as the reddening of the sun as it approaches the horizon and are well established. Pure water absorption was taken from Pope and Fry (1997), and the particle scattering phase function was an average-particle phase function based on measurements by Petzold (1977); tabulated values are given by Mobley [(Mobley 1994), table 3.10]. Chlorophyll fluorescence was calculated from chlorophyll-a concentration using a modeled phytoplankton absorption spectrum taken from Prieur and Sathyendranath (1981) and a fluorescence efficiency of 0.02 that was independent of excitation wavelength. Inelastic Raman scattering by the water molecules was also included (Gordon 1999).

The parameters for the visual system were as follows. The pupil sizes of the species were taken from the literature as previously described, using lens diameter as a proxy. Because temporal resolution has been measured for few oceanic teleosts or elasmobranchs, we estimated temporal resolution using a measure of critical flicker frequency (CFF) in humans (60 Hz; Mankowska et al 2021) for species with depth ranges up to 20 m; then, for each tenfold drop in light level, we reduced that starting temporal resolution value by 10 Hz until a depth of 300 m, where temporal resolution was leveled off at 5 Hz (Tyler and Hamer 1990). Ocular transmittance and quantum efficiency were set at typical values of $\tau$ = 0.8, and $\kappa$ = 0.34. We assumed, based on Warrant and Locket (2004), that the photoreceptors in the fovea were rods and had their peak absorbance at 480 nm, an absorption coefficient of 0.064 $\mu$ m^−1^, and outer-segment lengths of 50 $\mu$ m. The first two values are typical for deep-sea vertebrate eyes, but the length of photoreceptors is known to vary in deep-sea species. This and the lack of information about temporal resolution specifically in each of the species in our database were the two largest sources of uncertainty in parameterizing the model. Given the enormous range of photon catch values considered in the model, even substantial variation of these two factors would have little effect on the overall patterns. Additionally, the species in which temporal resolution have been measured are in relatively close agreement to those derived from our method above [e.g., swordfishes, (Fritsches et al. 2005); escolars, (Landgren et al. 2014)]. To account for the fact that minimum contrast thresholds in fishes (and indeed any animal) have never been measured to be below 0.005, the model did not allow it to go below this value. Of note is that the “optimal” visual acuity that we generate with our model is of course only optimal for the parameter values described above; however, specific parameter values were chosen because they are found in large portions of the daytime pelagic ocean, and thus are applicable to the natural habitats of a large number of species.

### Statistical analyses

To further explore the results of the optical model, we examined relationships between acuity, body length, and $\beta$ while controlling for phylogenetic relatedness, in several ways. First, we calculated the degree of dependence of each variable on phylogenetic history by calculating phylogenetic signal, represented by Pagel’s λ (Pagel 1999), using the phylosig function of the package *phytools* (Revell 2012). Pagel’s λ ranges from 0 (indicating complete independence between phylogeny and the distribution of a trait) to 1 (complete covariance between trait distribution and phylogenetic structure). We then used phylogenetic generalized least squares (PGLS) models to examine relationships between acuity and $\beta$; body length (represented by total length) and $\beta$; and acuity and body length. In these models, body length and $\beta$ were log10 transformed to improve normality. PGLS models were run using the package *phylolm* (Ho and Ane 2014).

Lastly, we fit a series of PGLS models in which acuity was the response variable, and all combinations of $\beta$, body length, and their interaction were predictors. We ranked models using the Akaike Information Criterion corrected for small sample sizes (AICc; Akaike 1974; Burnham and Anderson 2002), and assigned ΔAICc values by calculating the difference between the AICc value of the best-fit model (that with the lowest AICc value) and each other model. Following Burnham et al. (2011), ΔAICc values were used to calculate the relative likelihood for each model *i* using the formula *l*_*i*_ = exp[− (1/2) Δ _*i*_]. We then calculated the probability that a given model, w_*i*_, was the best model by dividing the likelihood of a given model by the sum of the likelihoods of all models (Burnham et al. 2011).

## Results

### Acuity database

**Fig. 3:**
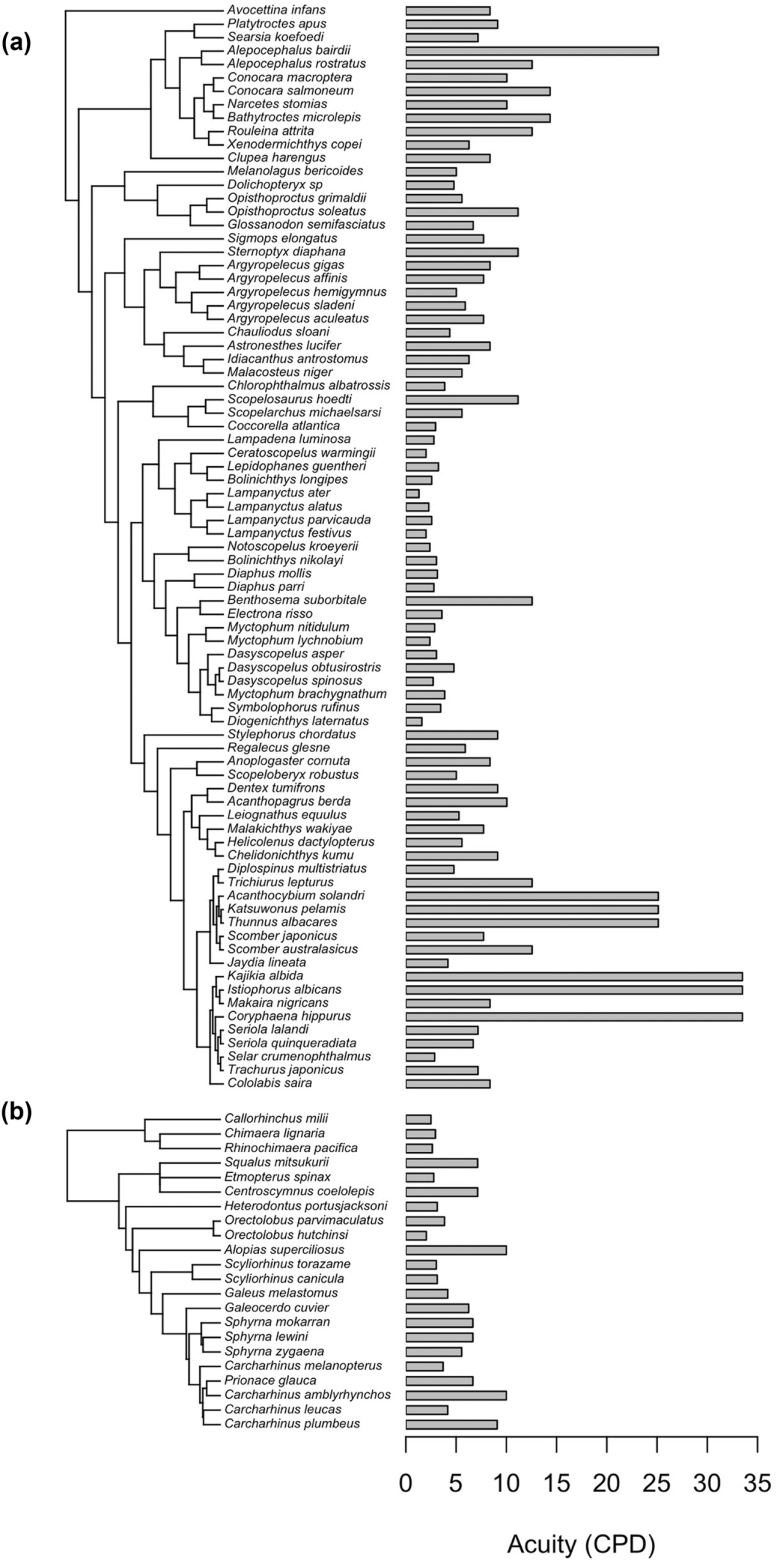
Reported visual acuity in cycles per degree in the 103 species for which we gathered acuity data from the literature, including **A** 81 species of teleost fishes and **B** 22 species of elasmobranchs. The tree in **A** was pruned from the Fish Tree of Life Project’s RAxML phylogram (Rabosky et al. 2018), and the tree in **B** was generated using a consensus tree derived from 100 phylogeny subsets from VertLife.org (Stein et al. 2018)

From the literature, we obtained data on acuity for 103 species of teleosts and elasmobranchs for which depth range data were also available (Fig. 3). After excluding those in benthic habitats and those for which no lens diameter data were available, the database consisted of 97 species (Table 1). For each species, we calculated the $\beta$ parameter for its maximum daytime depth, minimum daytime depth, and the midpoint of the depth range. However, we excluded from analyses any species with a $\beta$_Max_ (the $\beta$ value calculated from its minimum depth from the surface) value of < 10^4^ photons per steradian because sighting distances under ambient light are essentially zero at such $\beta$ values. Thus, the final comparison with predicted acuity used data from 82 species: 64 species of teleost fishes, for which acuity (mean ± standard deviation) was 8.1 ± 7.5 cpd, and 18 species of elasmobranchs, for which acuity was 5.4 ± 2.5 cpd. Minimum daytime depth ranged from 0 to 800 m (mean ± standard deviation: 203 ± 206 m), and maximum daytime depth ranged from 12 to 2000 m (mean ± standard deviation: 580 ± 517 m).

### Model results

**Fig. 4:**
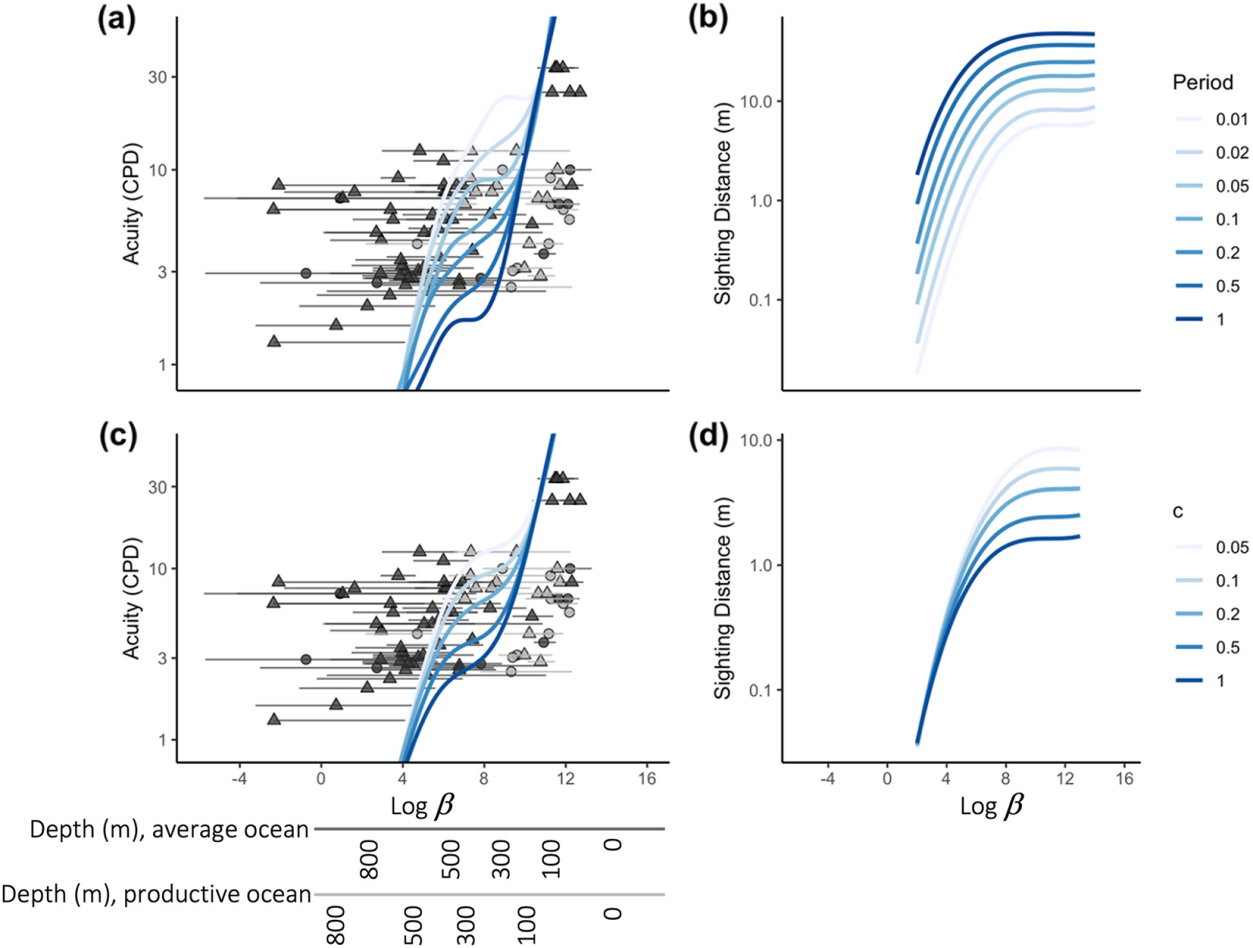
Measured acuities (points with horizontal black and gray lines) and model predictions (smooth curved lines) across variation in $\beta$ (photon catch by one RGC per steradian) (**A, C**), and sighting distances for predicted optimal acuity across variation in $\beta$ (**B, D**), for different spatial periods (in meters) of the target stripes being viewed (**A, B**), or for different attenuation coefficients *c* of the water (**C, D**). For (**A, B**), stripe contrast was 0.6 (80% and 20% reflective white and black stripes, respectively) and the attenuation coefficient *c* was 0.05 m^−1^, and for (**C, D**), stripe contrast was again 0.6 and stripe period was 2 cm. Point and line shading show species that do (light gray) and do not (dark gray) live in or under productive coastal or northern polar water, and secondary axes translate $\beta$ into depth values for each water type assuming the median pupil diameter of the animals sampled (4.3 mm) and an integration time of 0.25 s. Points represent the $\beta$ value resulting from the midpoint of a species’ daytime depth range, with the horizontal lines extending between the $\beta$ values calculated from minimum and maximum depth. Triangles represent teleost fishes, circles represent elasmobranchs

In the case where the period of the stripes was varied from 0.01 to 1.0 m (with the attenuation coefficient of the water always remaining at the typical ocean value (at 480 nm) of 0.05 m^−1^) (Fig. 4a, b), the predicted optimal acuity depended strongly on stripe period for 10^4^ < *β* < 10^10^ (depth range ~ 150–600 m for viewers with 4.3-mm pupils—the median for the database). For brighter/shallower and deeper/darker depths, predicted optimal acuity was roughly independent of stripe period, but strongly dependent on depth. Predicted optimal acuity dropped from 1 cpd for *β* = 10^4^ (depth ≅ 600 m) to 0.1 cpd for *β* = 10^2^ (depth ≅ 1000 m) and reached up to ~ 100 cpd for surface *β* values of ~ 10^12^. The predicted maximal sighting distances that corresponded to these predicted optimal acuities also depended strongly on both stripe period and depth, ranging from ~ 2 cm for discerning a 2-cm wide stripe pair at ~ 1000 m depth to 50 m for discerning a 1-m wide stripe pair at the surface. For *β* > 10^10^ (depth <  ~ 150 m), predicted maximum sighting distance was nearly independent of depth but still dependent on stripe period.

In the case where the attenuation coefficient of the water varied from 0.05 m^−1^ (typical 480 nm value for the deep sea) to 1.0 m^−1^ (exceedingly turbid near-shore water), with the stripe period always remaining at 2 cm (Fig. 4c, d), the predicted optimal acuity also depended on water turbidity for 10^4^ < *β* < 10^10^. For brighter/shallower and deeper/darker depths, predicted optimal acuity was roughly independent of turbidity, but again strongly dependent on depth and in the same fashion as seen for the stripe period predictions. The predicted maximal sighting distances that corresponded to these predicted optimal acuities depended strongly on both turbidity and depth, ranging from ~ 3 cm for all water turbidities at ~ 1000 m depth to 8.5 m for viewing the 2 cm stripe pattern in the clearest water at the surface. For *β* > 10^10^ (depth <  ~ 150 m), predicted maximum sighting distance was nearly independent of depth but still dependent on water turbidity.

Although the large depth range of many of the species and the uncertainty about the period of the patterns they might be viewing precluded exact comparisons, the measured acuities of the animals with mid-depth *β* values greater than 10^4^ (daytime depth <  ~ 600 m in clear oceanic waters) were typically within a factor of ten of the predicted optimal acuities, with the large majority being with a factor of three. The measured acuities of species with mid-depth *β* values greater than 10^10^ (depth <  ~ 150 m) were all less than predicted, while the measured acuities of species with mid-depth *β* values less than 10^4^ (depth >  ~ 600 m) were all more than predicted. At even greater/darker depths, the measured acuity became much greater than predicted, in some cases greater than 100 times the predicted value. At epipelagic depths (depth < 200 m; *β* >  ~ 10^9^), the measured acuity in oligotrophic waters (21 ± 12 cpd) was greater than that in productive waters (6.0 ± 2.9 cpd) (p < 0.005; two-tailed *t *test assuming unequal variances).

**Fig. 5:**
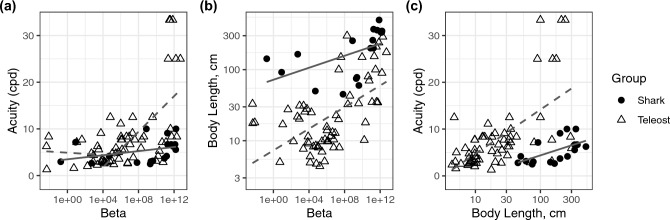
Relationships between **A** acuity and $\beta$; **B** body length and $\beta$; and **C** acuity and body length in sharks (black circles; solid lines) and teleost fishes (open triangles; dashed lines). In Panel (**A**), separate lines have been fitted for teleosts with $\beta$ values greater than and less than 10^4^. Lines represent linear model fits, and gray shaded areas represent confidence intervals

To further explore the model results, we then statistically explored how both phylogenetic relatedness and body length related to the $\beta$ parameter. Correcting for phylogenetic relatedness necessitated examining elasmobranchs and teleosts separately. However, given low sample sizes for elasmobranchs, statistical analyses were not meaningful; therefore, although we visually present the data for elasmobranchs alongside those for teleosts (Fig. 5), statistical analyses were performed only on data for teleost fishes.

First, we found significant phylogenetic signal, as represented by Pagel’s *λ*, in both acuity (*λ* = 0.23, *p* = 0.005) and body length (*λ* = 0.43, *p* = 0.0001), but not in $\beta$ (*λ* = 0.02, *p* = 0.77). Thus, both acuity and body length significantly covary with phylogenetic structure, but $\beta$ (and thus depth) does not.

Phylogenetically corrected (PGLS) models showed that $\beta$ is a significant predictor of acuity (*p* < 0.0001; Fig. 5A). We also found that $\beta$ is a significant predictor of body length (*p* = 0.01; Fig. 5B); thus, as depth increases (and thus $\beta$ gets smaller), fish body lengths get smaller as well. This likely arose not because of a real-world trend of decreasing body lengths with depth (across all species), but rather as a result of sampling bias given the challenge of capturing large deep-sea organisms. Lastly, PGLS models showed that body length is a significant predictor of acuity (*p* < 0.0001; Fig. 5C), as expected given known correlations between acuity and eye size, and in turn tight correlations between eye size and body length, in fishes. Interestingly, visually examining the data for elasmobranchs alongside teleost fishes (Fig. 5) showed that for a given body length, sharks have much lower acuity than do teleost fishes.

Lastly, we built a PGLS model in which acuity was predicted by body length, $\beta$, and their interaction. AIC-based model selection (Table 2) showed that the best-fit model included all three of these terms as co-predictors; support for this best-fit model, as indicated by model probability, was high (*w* = 0.93). The next best-fit model included both $\beta$ and length as co-predictors, but was a substantially worse fit for the data than the best model, with a ΔAIC of 5.62 and a model probability of only 0.06. Importantly, models with any combination of our parameters were a better fit than a null model (Table 2).

**Table 2:** Summary of predictor combinations of variables fitted to phylogenetic generalized least squares models of acuity and resultant Akaike Information Criterion (AICc) scores

Model	AICc	ΔAICc	Model weight
$\beta$ + length + $\beta$*length	402.0	0	0.93
$\beta$ + length	407.5	5.51	0.06
Length	410.3	8.33	0.01
$\beta$	419.1	17.1	0.00
Null model	431.3	29.4	0.00

*Indicates an interaction term

## Discussion

Here, we built a visual/optical model that predicted the optimal acuity (for a set of parameters that represent the daytime clear, open-ocean waters that cover a large part of our planet’s surface) for resolving patterned stimuli and compared it with a database of actual acuities measured using anatomical parameters in teleost fishes and elasmobranchs. We found that the actual visual acuity of epipelagic and upper mesopelagic species was fairly well predicted by our model of optimal acuity, but that species living in the deeper mesopelagic (> 600 m depth) had far higher acuities than predicted.

These results are consistent with the prediction that animals found at epipelagic and upper mesopelagic daytime depths have visual acuity optimized for resolving patterns, while species found in the mid-meso-to-bathypelagic have vision optimized for best locating point sources, types of visual specialization that align with the types of visual scenes that animals in each depth range view (Warrant 2000; Warrant and Locket 2004). At depths shallower than about 600 m, visual scenes are extended, and animals may use their visual systems during the day to resolve a variety of objects, whereas at daytime depths greater than 600 m (and at shallower depths at night), animals operate in a visual world that consists primarily of bioluminescent point sources. This is consistent with prior research showing that eyes in the deep sea have high acuity in specific parts of the visual field (Wagner et al. 1998), and that these sharp foveae can help animals localize point sources at ecologically relevant distances (Warrant 2000).

Overall, the results suggest that the visual acuity of fishes is depth dependent and under selection for detecting either patterns or point sources, even after correcting for the fact that sampling bias leads to smaller animals (and thus lower acuities) at deeper depth. Selection for detecting and localizing point sources should be strong in deep-sea fishes, because bioluminescent flashes are often indicative of resources, which are rare in the deep sea. Missing a flash could mean failing to take advantage of a potential food item or losing out on a mating opportunity (Warrant 2000).

Interestingly, the highest acuities observed in this study were not found in deep-dwelling fishes, but were from epipelagic species, specifically large predatory open-water species (mahi-mahi *Coryphaena hippurus*, sailfish *Istiophorus albicans*, marlin *Kajikia albida*, wahoo *Acanthocybium solandri*, and the tunas *Katsuwonus pelamis* and *Thunnus albacares*). Thus, it may be that some selective forces present in the open-ocean euphotic zone, for example selection pressures associated with high energy demand in an extremely patchy prey environment, can drive acuity to be the highest known in fishes (Caves et al. 2017), even higher than is necessary in the deep sea for localizing point sources.

### Limitations of the acuity database and model

One limitation of this study is that, to maximize the available sample size for sparsely sampled deep-sea species, we used acuity that had been measured using either the density of the photoreceptors or the density of the RGCs, which do not always yield identical estimates of acuity. At least in teleost fishes, however, since foveae are specializations for high acuity, it is reasonable to assume that the ratio of cone photoreceptors to RGCs is 1:1 at the foveal center (e.g., Fritsch et al. 2017), and since papers that report acuity do so by calculating it from the area of highest density (the fovea), these methods likely yield similar estimates in a given species. Of note, however, is that foveae have been reported in deep-sea teleosts, but not elasmobranchs (Warrant and Locket 2004). Additionally, we also included measures of acuity generated using lens optical quality. Although studies of shallow-water fishes have sometimes found optical lens quality to overestimate acuity (e.g., Charman and Tucker 1973), one study of acuity in pelagic fishes found that the resolving power of the lens is a relatively good match for the resolution provided by the retina (Gagnon et al. 2013).

Additionally, channel density (whether photoreceptor or RGC) can vary widely across different parts of the eye, meaning that parts of the visual field can be perceived more sharply than others (Warrant and Locket 2004). However, in the vast majority of acuity studies, the highest recorded acuity from anywhere in the eye is reported as the acuity for a species, and variation within an eye or across individuals is ignored. In line with this, in our analysis, we used a single value for each species. Thus, open questions remain regarding precisely how acuity is optimized at various depths, in terms of whether large portions of the visual field, or only select portions, can change to resolve patterns or point sources as light levels drop.

As previously stated, two limitations of the model are: (1) that the temporal resolution of oceanic teleosts and elasmobranchs at the relevant light levels has seldom been measured, and (2) the rod-outer-segment lengths of these same animals are also poorly known. Fritsches et al. (2005) measured the flicker-fusion frequency (inverse of Δ*t*) of the swordfish (*Xiphias gladius*) and found that it dropped in a log-linear fashion from 40 Hz at 100 m to 2 Hz at 500 m in clear oceanic waters. Landgren et al. (2014) found a similar log-linear drop in flicker-fusion frequency with depth in the deep-sea escolar (*Lepidocybium flavobrunneum*), dropping from 10 Hz at the surface to 2 Hz at 500 m. The temporal resolutions modeled in this paper are in the same range and follow the same log-linear relationship between light level and flicker-fusion frequency. Regarding limitation (2), we used a rod-outer-segment length of 50 μm. Although some deep-sea fishes have longer rod outer segments, a 50 μm length already results in 96% absorption of incident light at the dominant wavelengths of deep-sea light (480–490 nm). Thus, ignorance of the rod-outer-segment lengths likely had little effect on the predictions of the model, since a longer outer-segment length (as found in some deep-sea fishes) would not increase absorption by much.

A larger issue is that many of the species sampled had large daytime depth ranges that resulted in enormous variation in light level. Published depth ranges of oceanic species, because they often run from the deepest specimen ever sampled to the shallowest without noting the typical ranges at where the animals are found, tend to overestimate depth range and thus range of daytime light levels. We used “typical depth ranges” when known, but this was only possible for 1/3 of the species. Additionally, although we focused on daytime depth ranges, some fishes vertically migrate, and how nighttime depth ranges relate to visual function should be investigated in a future study. In general, however, nighttime depth ranges are much less often reported than daytime depth ranges.

A final limitation is the fact that sampling bias has limited the collection of fishes to smaller species at depth. For various reasons, it is challenging to catch large deep-sea species. Because acuity is known to be tightly correlated to eye size and thus body length (Caves et al. 2017), it could be that the drop in acuity with depth seen here is entirely due to the smaller sizes of collected deep-sea species. It is for this reason that we examined how well models including $\beta$ and body length fit our acuity data, using established and formal model selection techniques. These methods showed that the best model was one in which acuity in these species depended on both depth and body length (and their interaction).

## Conclusion

In conclusion, the acuity of the teleost fishes sampled depended on depth, even when the relationship between fish size and depth was accounted for. It decreased with depth—roughly matching a visual detection range model—until a depth of approximately 600 m, at which point acuity did not decrease further. At depths below this point, acuity rapidly became much higher than predicted by the visual detection range model, suggesting that acuity was not specialized for seeing patterns under ambient light, but instead for localizing the point sources of bioluminescence that are the predominant form of light at this depth. Extending this work further will require not only more species, but also tighter daytime depth range values.

## Data Availability

All data and codes necessary to produce the figures and statistical analyses are available in the publicly-accessible Dryad Data repository (Caves et al. 2023), https://doi.org/10.25349/D9K330.
